# A Novel High-Content Immunofluorescence Assay as a Tool to Identify at the Single Cell Level γ-Globin Inducing Compounds

**DOI:** 10.1371/journal.pone.0141083

**Published:** 2015-10-28

**Authors:** Marta Durlak, Cristina Fugazza, Sudharshan Elangovan, Maria Giuseppina Marini, Maria Franca Marongiu, Paolo Moi, Ivan Fraietta, Paolo Cappella, Gloria Barbarani, Isaura Font-Monclus, Mario Mauri, Sergio Ottolenghi, Fabio Gasparri, Antonella Ronchi

**Affiliations:** 1 Department of Biology, Nerviano Medical Sciences S.r.l., Nerviano, Milano, Italy; 2 Dipartimento di Biotecnologie e Bioscienze, Università degli studi di Milano-Bicocca, Milano, Italy; 3 Istituto di Ricerca Genetica e Biomedica del Consiglio Nazionale delle Ricerche, Cagliari, Italy; 4 Dipartimento di Sanità Pubblica, Medicina Clinica e Molecolare, Università degli Studi di Cagliari, Cagliari, Italy; 5 Dipartimento di Scienze della Salute, Università degli studi di Milano-Bicocca, Monza, Italy; Southern Illinois University School of Medicine, UNITED STATES

## Abstract

The identification of drugs capable of reactivating γ-globin to ameliorate β-thalassemia and Sickle Cell anemia is still a challenge, as available γ-globin inducers still have limited clinical indications. High-throughput screenings (HTS) aimed to identify new potentially therapeutic drugs require suitable first-step-screening methods combining the possibility to detect variation in the γ/β globin ratio with the robustness of a cell line. We took advantage of a K562 cell line variant expressing β-globin (β-K562) to set up a new multiplexed high-content immunofluorescence assay for the quantification of γ- and β-globin content at single-cell level. The assay was validated by using the known globin inducers hemin, hydroxyurea and butyric acid and further tested in a pilot screening that confirmed HDACs as targets for γ-globin induction (as proved by siRNA-mediated HDAC3 knockdown and by treatment with HDACs inhibitors entinostat and dacinostat) and identified Heme-oxygenases as novel candidate targets for γ-globin induction. Indeed, Heme-oxygenase2 siRNA knockdown as well as its inhibition by Tin protoporphyrin-IX (TinPPIX) greatly increased γ-globin expression. This result is particularly interesting as several metalloporphyrins have already been developed for clinical uses and could be tested (alone or in combination with other drugs) to improve pharmacological γ-globin reactivation for the treatment of β-hemoglobinopathies.

## Introduction

Sickle cell anemia (SCA) and β-thalassemia are among the commonest inherited diseases in humans, with more than 300,000 affected children born every year and with an estimated worldwide population of tens of millions patients suffering from these disorders [1]. The number of these patients is increasing because of the decreased mortality from nutrition problems and infections in the developing countries [2–4]. SCA is caused by a missense mutation within the adult β-globin chain. Hemoglobin tetramers bearing this altered β chain (HbS) tend to polymerize within the Red Cell, under hypoxic conditions, conferring the typical sickle shape, leading to cell lysis, small vessel occlusion, pain crises and organ damage. In β-thalassemia, the reduced synthesis of β chains causes unbalanced accumulation of α-globin that precipitates, resulting in ineffective erythropoiesis and anemia [5]. Coinheritance of Hereditary Persistence of Fetal Hemoglobin (HPFH), a condition where the expression of the fetal *HBG1/2* is maintained postnatally, can ameliorate β-globinopathies, by reducing sickle hemoglobin polymers in SCA and the α/non-α chain imbalance in β-thalassemia[6]. This observation led to the intensive search for fetal hemoglobin (HbF) inducers that could mimic the beneficial effects observed in HPFH[7–9]. Genome-wide association studies identified three major gene loci (Xmn1-HBG2, HBS1L-MYB and BCL11A) accounting for the majority of inherited HbF variance[10] but their exploitation as therapeutic targets is still distant. Another line of research focused on the development of drugs acting on γ-globin regulatory molecules: different classes of drugs (cytotoxic agents, HDAC inhibitors, DNA methyl transferase inhibitors) have been tested as HbF inducers but, despite the enormous effort in this direction and some encouraging results on some patients, no universal effective drugs have been found so far. Among them, hydroxyurea (HU) has been approved by the FDA for the treatment of SCA and has been recently considered for β-thalassemia, but its efficacy varies among patients. Indeed, about half of the patients do not reach therapeutic levels of HbF at HU doses of acceptable toxicity[11,12]. Other agents, such as short-chain fatty acids (Butyrate and its derivatives), 5-azacytabine, Decitabine and Tranylcypromine act on the epigenetic regulation of HbF, by inhibiting histones deacetylation or methylation of the *HBG1/2*, but their efficacy is still limited to a minority of patients[13–16].

These observations point to the strong need to identify new compounds stimulating γ-globin expression.

With this goal in mind, we set up a high-content screening platform based on multiplexed imaging on a variant K562 cell line (β-K562) spontaneously expressing significant levels of β-globin. Simultaneous analysis of DNA content, adult hemoglobin HbA (α2β2) and fetal hemoglobin HbF (α2γ2) resulted in a robust and sensitive assay, capable of detecting changes at the single cell level in hemoglobinization and in γ/β ratio in response to drugs, as proved by the response of β-K562 to the known γ-globin inducers hemin, hydroxyurea and butyric acid and to two additional HDAC inhibitors: entinostat and dacinostat.

The method was further validated by transfecting β-K562 with a panel of 70 siRNAs. Among them, we identified HMOX2, coding for Heme-oxygenase2 (HO-2), as a gene whose knockdown greatly increases γ globin levels, both in terms of percentage of expressing cells and of γ-globin accumulation per cell. Tin Protoporphyrin IX, a prototypical compound inhibiting HO-2, induced selective γ-globin accumulation in β-K562, suggesting that Heme-oxygenases could be a promising pharmacological target to ameliorate the α/β chains unbalance in β-hemoglobinopathies.

## Materials and Methods

### Cell lines and chemical treatments

ECACC-K562 (European Collection of Cell Cultures) and β-K562 (a kind gift of Prof. G. Ferrari, HSR, Milano) were grown in standard conditions[17]. β-K562 were originally purchased from ATCC (CCL-243™). Doubling times were calculated on cells growing in exponential phase. β-K562 authentication was obtained by short tandem repeat fingerprinting (AmpFlSTR Identifiler Plus PCR Amplification kit -Applied Biosystems-), as described in [18]. For chemical treatments, 5 x 10^4^ cells were exposed to increasing doses in 24-well plates. After four days, cells were analyzed by RTqPCR or high-content analysis. All experiments were performed in triplicate (at least two technical replicates per experiment). Chemicals and antibodies are listed in S1 Table.

### siRNA oligonucleotide transfections

β-K562 cells were transfected with siRNA oligonucleotides (H-Silencer Select Druggable Genome siRNA Library V4, Ambion). A siRNA oligo targeting the proteasome subunit PSMC3 and a non-targeting oligo (siNTO) were used as positive and negative controls for transfection (siRNA sequences are listed in S2 Table). At least two siRNAs oligonucleotides per gene were transfected by using lipofectamine® RNAiMAX (Invitrogen), as described in S1 File.

### Immunofluorescence and high-content analysis

Cells were collected and fixed in 3.7% paraformaldehyde for 20’ at RT, washed and permeabilized in staining buffer (PBS with 0.05% v/v Triton® X-100 and 1% w/v powdered milk) for 30’. After washing in PBS, cells were incubated overnight at 4°C in staining buffer containing the appropriate antibodies and 1μg/ml Hoechst 33342. After washing, cells were resuspended in PBS and transferred to 96-well CELLSTAR®, Black/μClear® plates (Greiner Bio-One). Plates were spun for 5’ at 2g to facilitate cell attachment, sealed and analyzed with the ArrayScan VTI high-content screening reader (Thermo-Fisher Scientific). At least 600 cells were acquired in each well with a 20x magnification in three fluorescence channels (blue, green and red). The Molecular Translocation Bioapplication was used to determine the cell count per field, the nuclear area and intensity (based on the Hoechst staining in the blue channel) and the cytoplasmatic fluorescence intensity of β (green) and γ (red) globins. For simultaneous globins/GlycophorinA staining, APC-anti-CD235 antibody was added for 2 hours to cells already stained for globins.

### Confocal microscopy

K562 and β-K562 cells, stained as above, were transferred to a microscope glass slide and mounted with Mowiol (Sigma-Aldrich). Microphotographs were acquired with a confocal Zeiss microscope LSM710.

### Flow cytometry

10^6^ cells were washed, fixed and permeabilized for 10’ on ice, then washed and incubated in PBS+1% milk for 20’. After washing, cells were stained overnight at 4°C in PBS+1% milk containing the appropriate antibodies. After washing, cells were analyzed with FACSCalibur (Becton Dickinson).

### RNA Isolation and RT-PCR

Total RNA from 10^6^ cells was extracted with TRI Reagent (Applied Biosystems), treated with RQ1 DNase (Promega) for 30’ at 37°C and retrotranscribed (Applied Biosystems). Negative control reactions (RT^-^) gave no signal. Real time analysis was performed using ABI Prism 7500, (Applied Biosystems). Primers are listed in S3 Table.

### Statistical analysis

Each experiment was statistically analyzed using a paired, two tailed Student-*t*-test.

## Results

### Identification and characterization of a K562 variant subclone expressing β-globin

K562 are probably the most extensively used cellular model of a human “erythroid” cell. This line was established in 1975[19] from a patient with chronic myelogenous leukemia (CML) in blast crisis. K562 have been widely used to study the molecular regulation of embryonic and fetal globin genes and to assess the therapeutic potential of differentiation-inducing drugs[20]. However, the major limitation for their use in the study of the differential γ/β regulation is their “fetal-like” pattern of globin genes expression, since they exclusively express embryonic (HbGower-2, α2ε2) and fetal (HbF, α2γ2) hemoglobin[21,22].

While characterizing different K562 subclones, we came across a variant clone expressing the adult *HBB*, that we named β-K562. These cells are morphologically similar to ECACC (European Collection of Cell Culture) K562 cells, here considered as “prototypical” K562 cells (not shown). Moreover, K562 and β-K562 have a similar doubling time (S1A Fig) and are equally sensitive to drugs known to inhibit K562 proliferation: imatinib mesylate and dasatinib, two tyrosine kinase inhibitors targeting the bcr/abl fusion protein and doxorubicin, a DNA intercalating agent (S1B Fig). In addition, β-K562 fingerprinting characterization by using the STR AmpFlSTR Identifiler Plus, gave a profile substantially corresponding to K562 (Identity score[18] of 89.2%, not shown). Despite their *bona fide* “K562-like” profile, β-K562 do express the adult β-globin chain, as assessed by flow cytometry (FCM) analysis (S1C Fig).

Based on this observation, we reasoned that the β-K562 subclone could be used to set up an immunofluorescence high-throughput, high-content screening platform to search for new genes/drugs modulating hemoglobinization and, in particular, the γ/β ratio.

### Development of a multiplexed high-content assay for the quantification of γ- and β-globin content in β-K562 at the single-cell level

**Fig 1:**
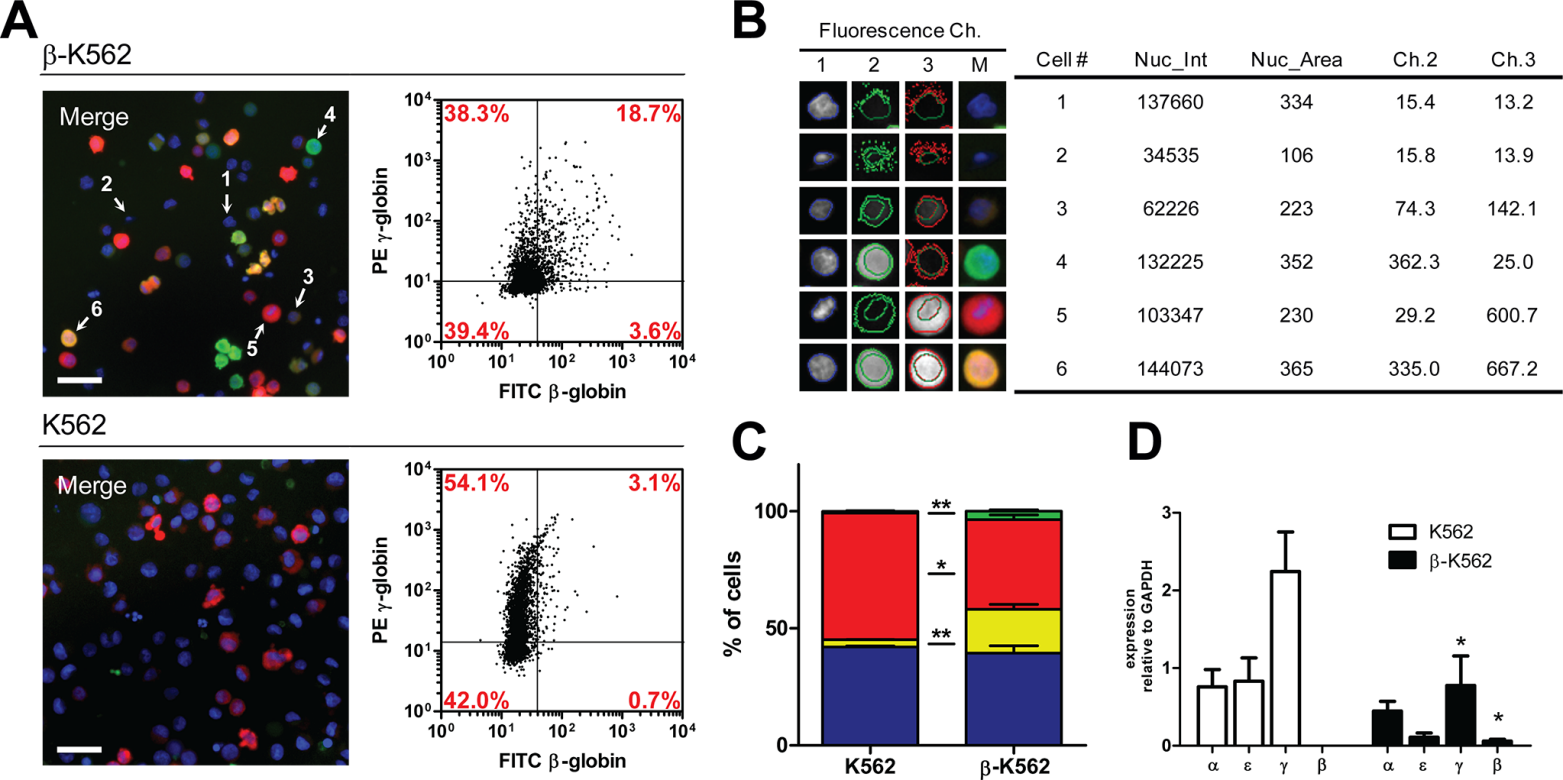
Analysis of γ/β globin levels by immunofluorescence and automated image capture. A) Image acquisition and analysis for β-K562 and K562. Merged signals of DNA (Hoechst-33342), β-globin and γ-globin are read in channel 1 (Ch1), channel 2 (Ch2) and channel 3 (Ch3), respectively (see also S1D Fig). Bar = 50μm. The intensity value of signals is automatically assigned by the instrument and converted into a corresponding intensity of color. The relative scatter plots show the distribution of double γ^-^β^-^ negative, single γ^+^β^-^ positive, single γ^-^β^+^ positive and double γ^+^β^+^ positive cells (x axis: FITC-β-globin; y axis: PE-γ-globin). Numbers within plots refer to the averaged percentage of cells within each population from three independent experiments (n = 3). The relative st.errors are shown in panel C: *p<0,05; ** p<0,01; ***p<0,001. B) Quantitative fluorescence imaging of single cells: cells numbered from 1 to 6 in panel A are taken as an example of γ^-^β^-^ double negative (1 and 2), single γ^+^β^-^ positive (5), single γ^-^β^+^ positive (4) and γ^+^β^+^ double positive (3 and 6). C) Statistical analysis (n = 3): γ^-^β^-^ cells; red: γ^+^β^-^ cells; yellow: γ^+^β^+^ cells; green: γ^-^β^+^ cells. D) RTqPCR on α, ε, γ- and β-globins. Histograms show the relative levels of expression normalized on glyceraldehyde-3-phosphate dehydrogenase (GAPDH). n≥3, statistical analysis: *p<0,05; **p<0,01; ***p<0,001.

5x10^4^ K562 or β-K562 were seeded in 24-well plates. Nuclei were stained with Hoechst-33342; γ- and β-globins were immunostained by using specific PE-anti γ and FITC-anti β-globin antibodies, respectively (S1D Fig). Cells were subsequently analyzed with an Array Scan VTI reader (Thermo-Fisher Scientific) and data were acquired and processed as shown in Fig 1A and 1B to obtain an automated and quantitative fluorescence imaging at a single cell level. The intensity of the staining is automatically converted in the corresponding intensity of colors: blue for Hoechst, green for β-globin and red for γ-globin.

The detection threshold for the scoring of single γ^**+**^-, β^**+**^- and double β^**+**^γ^**+**^-cells was defined by using cells stained with the respective isotype controls (PE-IgG_1_ and FITC-IgG_1_, an example is shown in S1E Fig). When signals from the three single channels are merged (Fig 1A), the double expression of γ plus β results in an orange/yellow color of different intensity, depending on the amount of γ and β chains (see cells 3 and 6 in Fig 1B). This analysis allows measuring of both the percentage of single-positive (γ^+^ or β^+^) and of double positive (γ^+^β^+^) cells in each field. Moreover, the signal intensity per cell uncovers the intrinsic heterogeneity within the cell population. In a standard experiment, data are acquired from a minimum of 500 cells and plotted to give an immediate visual image of cell distribution with respect to globin accumulation per cell, as in Fig 1A, where a representative experiment is shown. The majority of K562 cells are γ^+^ (54.1% γ^+^β^-^ + 3.1% γ^+^β^+^), the remaining being mostly γ^-^β^-^, with just a few marginally β^+^ cells (3.1% γ^+^β^+^+0.7% γ^-^β^+^). In contrast, about 57% of β-K562 cells are γ^+^ (38.3% γ^+^β^-^ + 18.7% γ^+^β^+^) and about 22% of cells are positive for β staining (18.7% γ^+^β^+^ + 3.6% γ^-^β^+^). Moreover, the Mean Fluorescence Intensity (MFI, Y axis) of γ^+^ cells is higher than in β-K562 (Fig 1A and S1C Fig). The expression of β-globin in β-K562 was further confirmed by RTqPCR (Fig 1D). Of interest, an intrinsic heterogeneity of the culture is present also for β signal: the large majority of β^+^ β -K562 cells also co-express γ-globin (18.7% of total cell population, corresponding to ≈ 85% of β^+^ cells, Fig 1C), whereas only few cells appear to be completely “switched” to β expression (3.6% of total cell population, corresponding to≈ 15% of β^+^ cells, Fig 1C). Overall, these data confirm that β-K562 express β-globin and provide additional information on the population heterogeneity.

### Validation of γ/β globin high-content assay by using the known hemoglobin inducers hydroxyurea and butyric acid

**Fig 2:**
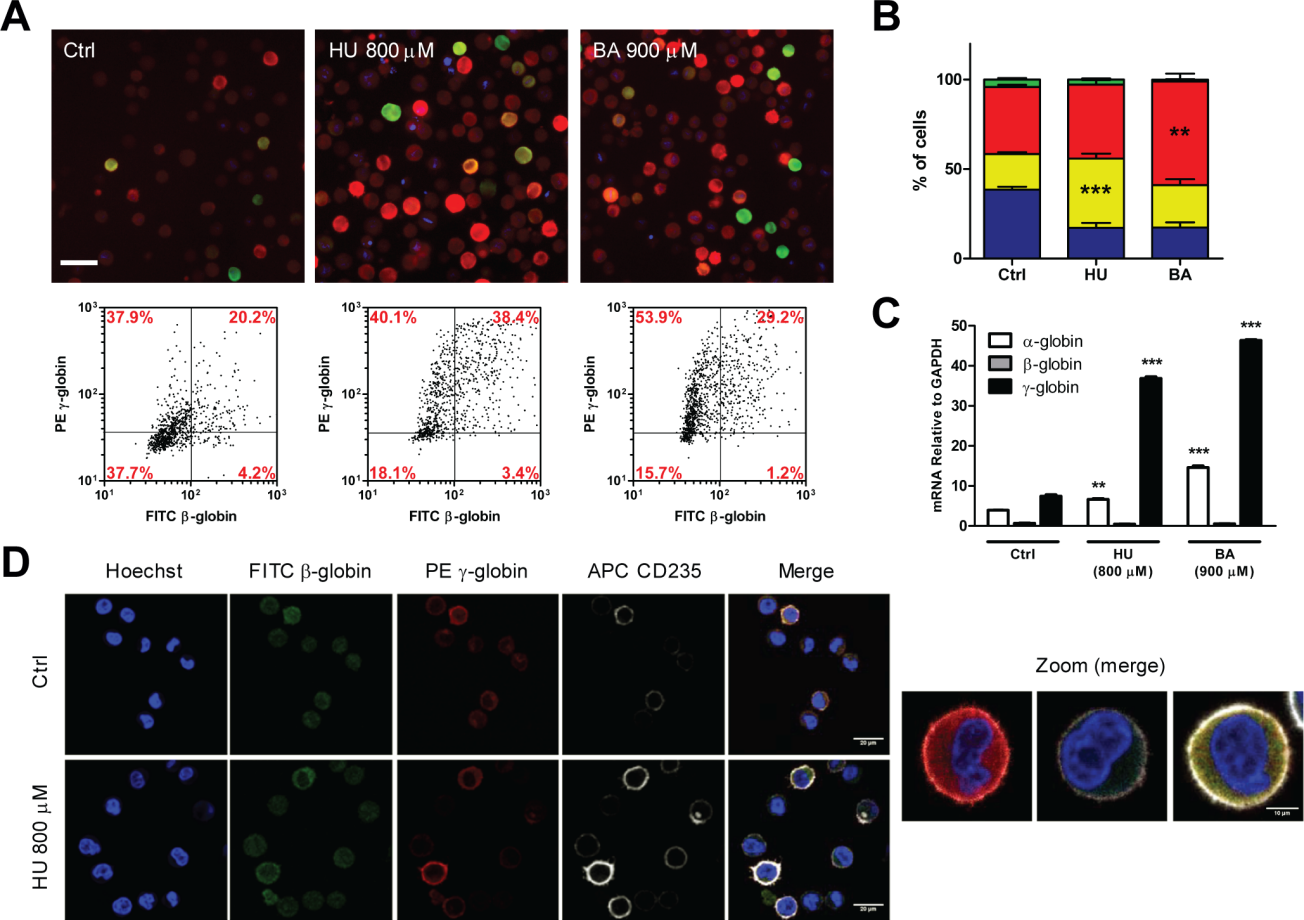
High-content analysis of compound-induced changes in globins accumulation. β-K562 cells were treated with 800μM hydroxyurea and 900μM butyric acid (n = 3, a representative experiment is shown here) and the same cells were analyzed in parallel by immunofluorescence and by RTqPCR 4 days after the addition of the drugs. A) Immunofluorescence images (Bar = 50μm) and relative scatter plots. Data from three independent experiments are presented and statistically analyzed (B) as in Fig 1. C) RTqPCR on α-, γ- and β- globins. Histograms show the relative levels of expression relative to GAPDH. D) Confocal analysis of β-K562 cells untreated or treated with HU as in panel A and subjected to a quadruple staining with Hoechst (blue), anti β- (green), anti γ-globin (red) and anti-CD235a (white). Magnification: 20x. Right panel: 40x magnification of individual cells γ^+^CD235a^+^ or β^+^CD235a^+^ double positive and γ^+^β^+^CD235a^+^ triple positive, respectively.

To test the sensitivity of the method in detecting changes of γ- and β-globin levels, we treated β-K562 cells with the known Hemoglobin inducers, hydroxyurea (HU) and butyric acid (BA). We measured the response of β-K562 cells at different pharmacological concentrations of these drugs (S2 Fig) and we analyzed the same cell samples by both RTqPCR and immunofluorescence. Fig 2 summarizes data relative to drug concentrations most commonly used in the literature to induce hemoglobinization in K562.

Both inducers, as expected, increase the hemoglobin content, reducing the number of double negative cells (Fig 2A and 2B). hydroxyurea induces both γ and β chains accumulation, as demonstrated by the increased percentage of γ^+^β^+^ double positive cells. Instead, butyric acid especially increases the percentage of γ^+^β^-^ cells, suggesting a different mechanism of action for these compounds (Fig 2B). In addition to increasing the number of globins-positive cells, these drugs also strongly increase the proportion of highly fluorescent cells (S2B Fig) acting predominantly on γ-globin accumulation versus β-globin.

At the mRNA level, both HU and BA stimulate γ expression by about 6–7 fold relative to untreated cells, whereas β-globin expression is essentially unchanged (Fig 2C). Finally, α-globin is moderately induced by both drugs, with BA eliciting the strongest increase (about four times). Overall, RTqPCR data confirm the effects observed at the protein levels, indicating the reliability of the assay.

The visual microscopy analysis can provide further information on additional parameters at the single cell level, such as nuclear morphology (Fig 2D) and/or the expression of specific markers of interest. As an example, the quadruple staining of β-globin, γ-globin, nuclei and GlycophorinA shows increased levels of GlycophorinA in β-K562 exposed to HU (Fig 2D).

### The transfection of a panel of siRNAs confirms the effectiveness of the immunomicroscopy platform to identify genes affecting hemoglobin synthesis and to test the efficacy of their modulators

We then tested the efficacy of our method in identifying genes that could affect hemoglobinization and/or γ/β ratio by siRNA transfection. Firstly, we set up different types of controls: i) as a negative control we transfected a non-targeting oligo (siNTO); ii) as a positive transfection control we targeted the proteasome 26S subunit, ATPase3 (siPSMC3), the knockdown of which should severely affect cell growth; iii) we knocked-down γ-globin (siHBG1) and β-globin (siHBB) obtaining almost complete ablation of the respective signals (S3A Fig).

**Fig 3:**
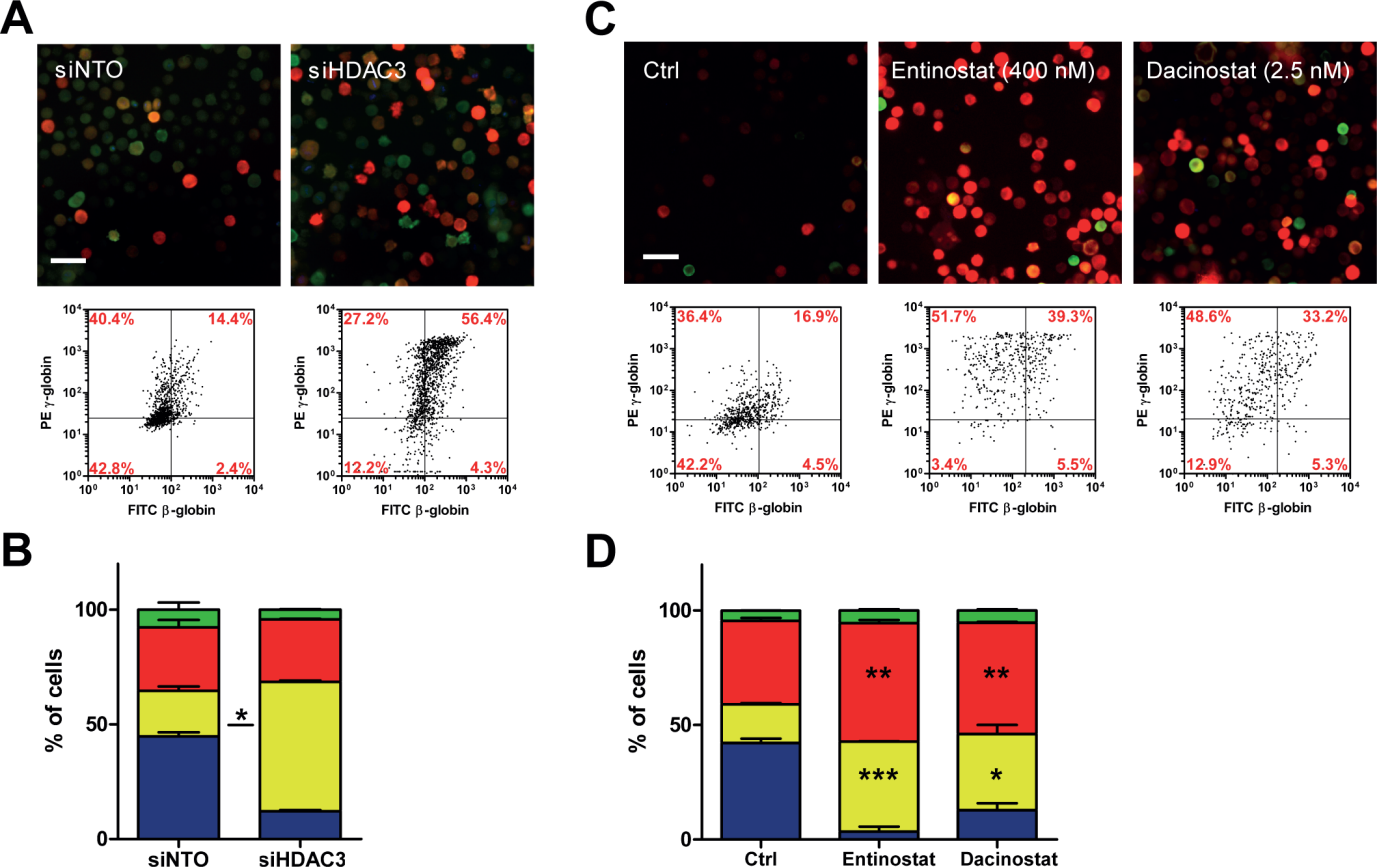
High-content γ/β globin analysis as readout of siRNA screening in β-K562 confirms HDAC as targets for γ-globin activation. A) Cells were transfected with a non-targeting oligo (siNTO) as negative control and with a siRNA directed to HDAC3. Two siRNAs were tested, with two technical replicates. C) β-K562 treated with two different HDAC inhibitors: entinostat and dacinostat (see also S3 Fig). A and C) Immunofluorescence images (Bar = 50μm) and relative scatter plots. Data from three independent experiments are presented and statistically analyzed (B and D) as in Fig 1.

To test the sensitivity in capturing changes in γ/β ratio, we knocked down HDAC3, reproducing the γ-globin promoter de-repression induced by treatment with butyric acid and its derivatives [23]. In HDAC3kd cells, we observed a marked increase in both γ- and β-globins, suggesting a broad mechanism of transcriptional de-repression (Fig 3A and 3B). In line with this, the cells treatment with HDAC inhibitors entinostat (MS-275) -an inhibitor of HDAC1 and HDAC3- and dacinostat (LAQ-824) induced γ-globin accumulation in a dose response manner (Fig 3C and 3D and S3B and S3C Fig). These results further confirm HDAC as targets for γ-globin reactivation and identify two additional inhibitors–in addition to BA (Fig 2)-, as potential therapeutic agents activating γ-globin.

### Is Heme-oxygenase a potential target for γ-globin induction?

**Fig 4:**
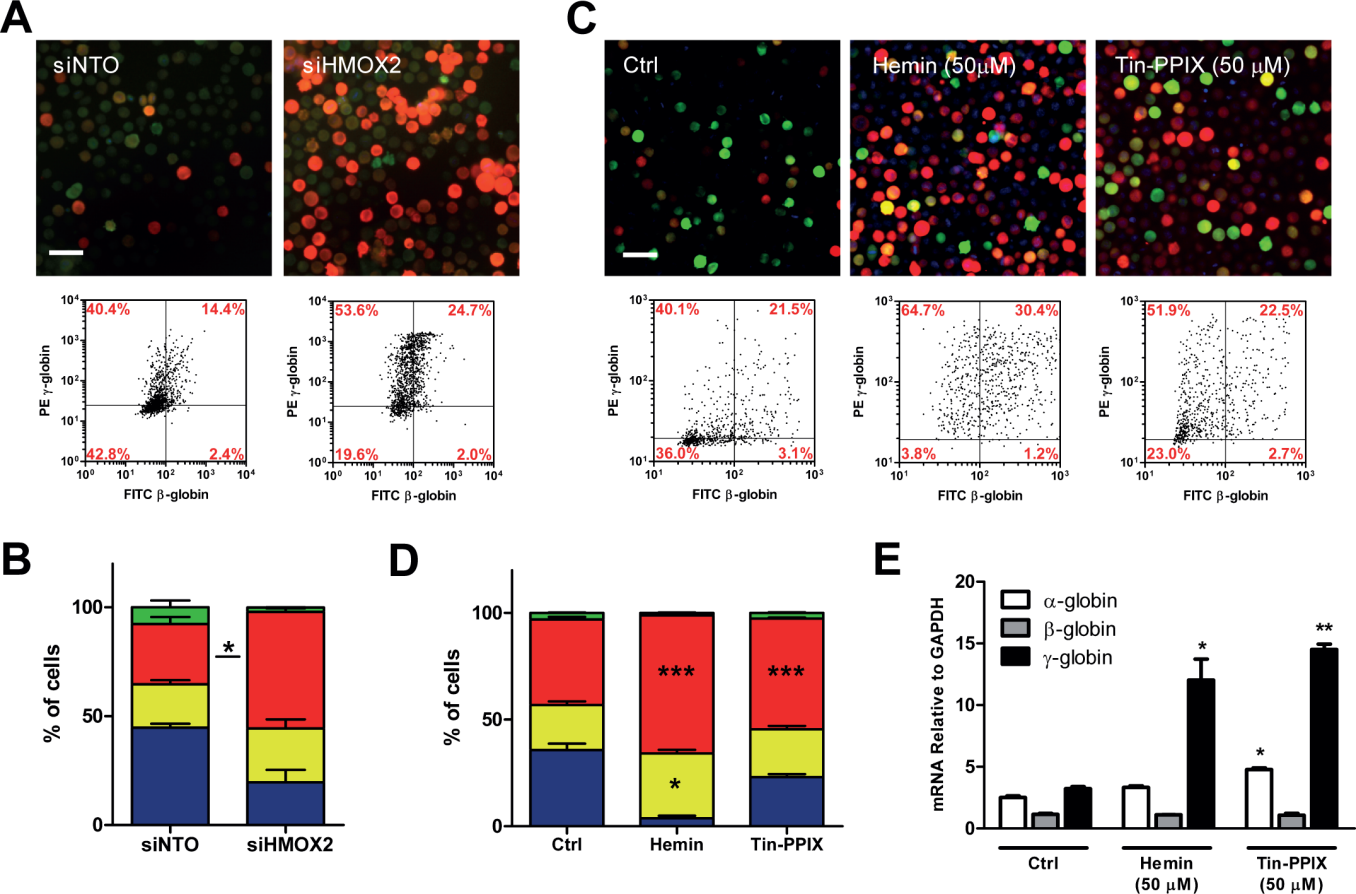
HMOX2 siRNA-mediated knockdown and hemin or Tin-PPIX treatment have similar effects on β-K562 hemoglobinization levels. A) Cells were transfected with a non-targeting oligo (siNTO) as negative control and with a siRNA directed to HMOX2. Two siRNAs (see also S4A Fig) were tested, with two technical replicates. C) Cells were treated with 50μM of either hemin or Tin-PPIX. A, C) Immunofluorescence images (Bar = 50μm) and relative scatter plots. Data from three independent experiments are presented and statistically analyzed (B and D) as in Fig 1. E) RTqPCR on α-, γ- and β-globins from cells treated with hemin or Tin-PPIX. Histograms show levels of globins expression relative to GAPDH (n = 3).

Given the above results, we undertook a pilot transfection screening on β-K562 with a panel of 70 siRNAs from the Ambion V4 library (S4 Table). Amongst them, siRNAs targeting the Heme-oxygenase (HO) coding gene HMOX2 (S4A Fig) gave a striking increase in globins with a prevalent accumulation of highly γ-globin expressing cells (Fig 4A and 4B). Heme-oxygenase catalyzes the conversion of heme to biliverdin (that, in turn, is immediately converted into bilirubin), iron and carbon monoxide. Both hemin, and Heme-oxygenase work on heme pool homeostasis, albeit in an opposite way, the first by replenishing the heme pool and the latter by promoting its degradation[24]. We then reasoned that the effect of the pharmacological inhibition of HO could result in globins stimulation similar to that known to be elicited by hemin (and possibly by the HMOX-2 knockdown shown in Fig 4). To test this hypothesis, we performed a dose/response treatment for hemin and Tin protoporphyrin IX (Tin-PPIX), here considered as prototypical HO inhibitor (S4B Fig). Fig 4C and 4D shows a striking increase in the percentage of γ-globin expressing cells and in γ-globin accumulation obtained upon Tin-PPIX treatment. Interestingly, whereas hemin treatment significantly increases the percentage of double γ^+^β^+^ positive cells, Tin-PPIX seems to have a more selective activity on γ, as confirmed by the MFI values (S4C Fig). Instead, at the mRNA level, the effect of hemin and Tin-PPIX are very similar (at equal concentrations), suggesting that post-transcriptional effects may play additional roles in balancing the α/non-α ratio.

## Discussion

We present a reliable and sensitive assay based on the unique property of β-K562 that express both γ and β genes to perform a first step high-throughput screening (HTS) to identify genes/drugs influencing the γ/β globin ratio. This overcomes the major limitation of the available human erythroid cell lines to study hemoglobin switching, i.e. their exclusive expression of embryonic/fetal genes. Indeed, HTS approaches published so far are almost exclusively based on cell lines transfected with a variety of reporters, under the control of γ and β globins promoters in the context of artificial genes/genomic arrangements[25],[26]. Recently established IPs-derived immortalized cell lines [27] represent a very promising tool for similar studies, but we feel that the easiness of growth and manipulation of β-K562 still represent a valuable advantage. Moreover, the growth factors independence of β-K562 prevents the possibility that globins expression could be influenced by subtle changes in growth conditions, and/or by other manipulations required to establish hES/hIPs.

More physiological models for these studies are also available: mice carrying artificial chromosome constructs encompassing the entire human β-locus, some of them containing knock-in fluorescent reporters under the control of globins promoters[28–30]. The advantage of such models with respect to cell lines is counterbalanced by their reduced manageability making them unsuitable for first-step HTS.

β-K562 cells are a valid tool for first-step screening because of their spontaneous expression of both γ- and β-globins from the intact β-locus (Fig 1). The presence of γ^+^β^+^ double positive cells and the plasticity in modulating γ and β expression suggest that in β-K562 the chromatin environment at the β locus is overall relatively accessible, making these cells particularly sensitive in detecting possible drugs/siRNA effects on γ activation. Regarding this issue, it is important to note that also in normal adult individuals there is a low proportion of HbF-positive cells–a few percent-, and immature cells in adults are known to transiently express γ-globin[31,32]. This suggests that a permissive environment for γ-globin expression may be present, although transiently, also in adult human cells, and could be modulated by drug treatment.

Specific antibodies allow a reliable picture of the final readout of interest, i.e. the amount of β- and γ-globin protein upon different drugs treatments/genes manipulations at the single cell level, providing hints about the heterogeneity of the response. In parallel, RTqPCR provides information on the differential regulation (transcription/RNA processing and stability versus translation) of globin expression (as well as on any other gene of interest) elicited by drugs/treatments (Figs 2–4) and modulators (siRNA targeting of HDAC3 and HMOX2, Figs 3 and 4).

The microscopy analysis can be further implemented to simultaneously analyze changes in multiple cellular parameters, such as morphology and/or expression of specific markers (Fig 3E), thus allowing the detection of possible effects of the tested drugs/treatments on different cellular processes. Importantly, the globin expression analysis is performed at the single cell level. This latter aspect is of particular relevance since the response to pharmacological agents that increase HbF is expected to involve either the accumulation of γ-globin in each single cell (due to transcriptional and/or translational effects) or the selection of subsets of erythroid differentiating “responder” cells, on the basis of a pre-existing heterogeneity.

β-K562 cells, allowed an efficient automated transfection-based screening, that led to the confirmation of HDACs as “druggable” targets for γ reactivation[16] (Fig 3) and to the identification of Heme-oxygenases (Fig 4) as possible novel target.

Regarding HDACs, we tested the effect on β-K562 of three different inhibitors: butyric acid (BA), a well known γ-inducing agent, entinostat (MS-275) and dacinostat (LAQ-824). Dacinostat has been considered for myeloid leukemia treatment because it promotes apoptosis in CML and AML cells [33]. Entinostat, an inhibitor of HDAC1 and HDAC3, is an inducer of cell differentiation in AML cells, not associated with apoptosis induction[34–36]. Whereas BA appears to specifically lead to γ-globin expression, entinostat and dacinostat lead to an increase in both γ- and β-globins, suggesting a different selectivity profile of these three compounds.

Concerning Heme-oxygenases, our study identified them as a novel class of potential druggable targets to reactivate γ-globin. Our results show that drug competitive inhibition of Heme-oxygenases by protoporphyrins (and thus presumably by other derivatives) is able to induce a substantial increase in γ-globin accumulation. In erythroid cells, the main function of heme is to serve as the oxygen-carrying moiety in hemoglobin (Hb), and heme biosynthesis is thus strictly coordinated with globin accumulation along with erythroid differentiation and maturation[37]. Heme, when present at high concentrations in the globin-unbound state (as in β-thalassemia), inactivates the heme-regulated eukaryotic initiation factor eIF2α-kinase (Heme Responsive Inhibitor, HRI), converting it into an inactive form. As active HRI inhibits the eIF2α translation initiation factor, excess heme increases globin synthesis, allowing a better balance between heme and globin chains[38]. Therefore, Heme-oxygenase inhibition, by increasing heme levels, is expected to favor globin synthesis. In addition, heme catabolism by oxidation is linked to generation of biologically active molecules, such as iron, biliverdin, CO and NO[24,37]. The observation that both heme addition and inhibition of HO increase globin expression in β-K562 is consistent with the expected role of heme in relieving translational inhibition by HRI; however, the increase of globin mRNA levels, particularly of γ-globin (mRNA and protein) is not easily explained only by a simple effect on globin mRNA translation. It is possible that the translational effect of HRI also operates on other factors, for example transcription or chromatin factors regulating the *HBG1/2*. Previously, studies of HMOX1 deficiency demonstrated a clear effect on both stress and steady state erythropoiesis[39–42], but less is known about the possible specific role of the constitutive HMOX2 (the most abundant isoform expressed by K562[43]) in erythroid cells[37,44]. Of interest, a polymorphism in the HMOX1 gene was associated with high levels of fetal hemoglobin in Brazilian patients with sickle cell anemia[45].

Both HO-1 and HO-2 are sensitive (albeit to a different extent) to the competitive inhibition elicited by different Metalloporphyrins and the availability of different drugs with different selectivity make HOs an attractive target for pharmacological inhibition. This result is of particular interest because different Metalloporphyrins have been developed and tested in clinics[46]. Our results suggest that their use, as single agent or in association with other known and FDA-approved HbF inducers, such as HU, should be explored as a promising tool to improve the α/non α globin chain imbalance in β-hemoglobinopathies. To address this question we are currently studying the effect of different Metalloporphyrins in mice carrying a complete human HBB locus transgene e and in *ex-vivo* cultures from thalassemic patients.

## Supporting Information

S1 Fig Characterization of the β-K562 subclone by comparison with ECAAC-K562. A) Growth curves (n = 2). B) Response (IC_50_) to imatinib mesylate, dasatinib and doxorubicin (n≥3). C) FCM analysis: cells were stained with anti γ- and anti β-globin antibodies and with the corresponding isotype controls and read in FL-1 (FITC, green channel) or in FL-2 (PE, red channel). A representative experiment is shown. **Immunofluorescence setup.** D) In the immunofluorescence analysis, nuclei were stained with Hoechst-33342; HbF and HbA were immunostained by using specific anti γ- and anti β-globin antibodies and signals were acquired in three single channels: blue for Hoechst (Ch1), green for β-globin (Ch2) and red for γ-globin (Ch3), respectively, and then merged for analysis (Merge). E) Images acquired in the single channels for representative isotype controls and relative scatter plots. Bar = 50μm.
(TIF)

S2 Fig Dose/response of β-K562 to Hydroxyurea and Butyric Acid. A) Representative ArrayScan pictures of β-K562 cells treated with increasing doses of HU and BA (n≥3). Bar = 50μm. B) Fluorescence intensity plots to better visualize the changes in mean fluorescence intensity (MFI) of stained cells upon drugs treatment. Y axis: number of events (cells); X axis: fluorescence intensity for β-globin signal (upper panels) or γ-globin signal (lower panels), respectively. Green/Red curves: treated cells. Black curve: untreated cells. The vertical dotted line within each panel corresponds to the threshold set in Fig 2A. The MFI and the percentage of positive cells (%) are indicated within each panel.
(TIF)

S3 Fig HDAC3 siRNA-mediated knockdown and HDAC inhibitors treatment in β-K562 confirm HDACs as targets for γ-globin activation. A) Cells were transfected with a non-targeting oligo (siNTO) as negative control and with a siRNA directed to PSMC3 as positive transfection control. As further control, siRNAs targeting γ- and β-globins greatly reduced the corresponding globins chains. For each gene, two siRNAs were tested, with two technical replicates (immunofluorescence images of representative experiments are shown). Bar = 50μm. Scatter plots are provided for each immunofluorescence image (n = 2). B) Representative ArrayScan pictures of β-K562 cells treated with increasing doses of entinostat and dacinostat. C) MFI plots as in S2 Fig.
(TIF)

S4 Fig Hemin and Tin-PPIX have similar effects on β-K562 hemoglobinization levels. A) HMOX2 knockdown: RTqPCR on cells transfected with a non targeting oligo (siNTO) and with two independent siRNA directed to HMOX2. B) Representative ArrayScan pictures of β-K562 cells treated with increasing doses of Hemin and or Tin-PPIX. Bar = 50μm. C) MFI plots as in S2 Fig.
(TIF)

S1 File siRNAs oligonucleotide detailed transfection method. (PDF)

S1 Table Chemicals and antibodies. (PDF)

S2 Table List of siRNA oligos. (PDF)

S3 Table List of primers used for RTqPCR. (PDF)

S4 Table List of genes tested by siRNA-mediated knockdown and selected from the Ambion-library. (PDF)
