# Supplementary figures and images for: A Novel High-Content Immunofluorescence Assay as a Tool to Identify at the Single Cell Level γ-Globin Inducing Compounds
Source: PLoS One. 2015 Oct 28;10(10):e0141083. doi: 10.1371/journal.pone.0141083 (PMC4624791; doi:10.1371/journal.pone.0141083)

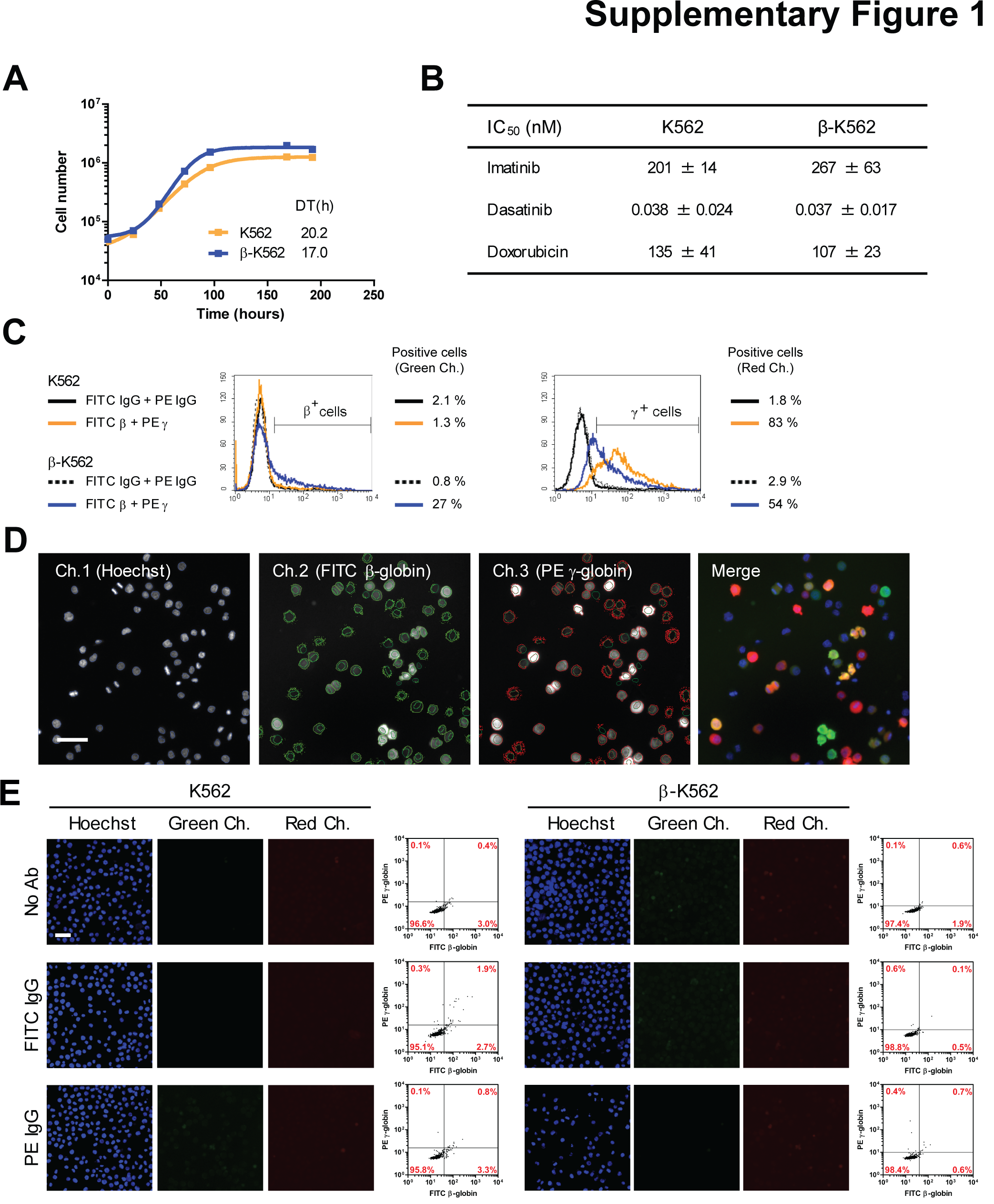

Supplement: S1 Fig — A) Growth curves (n = 2). B) Response (IC50) to imatinib mesylate, dasatinib and doxorubicin (n≥3). C) FCM analysis: cells were stained with anti γ- and anti β-globin antibodies and with the corresponding isotype controls and read in FL-1 (FITC, green channel) or in FL-2 (PE, red channel). A representative experiment is shown. Immunofluorescence setup. D) In the immunofluorescence analysis, nuclei were stained with Hoechst-33342; HbF and HbA were immunostained by using specific anti γ- and anti β-globin antibodies and signals were acquired in three single channels: blue for Hoechst (Ch1), green for β-globin (Ch2) and red for γ-globin (Ch3), respectively, and then merged for analysis (Merge). E) Images acquired in the single channels for representative isotype controls and relative scatter plots. Bar = 50μm. (TIF) [file pone.0141083.s001.tif]

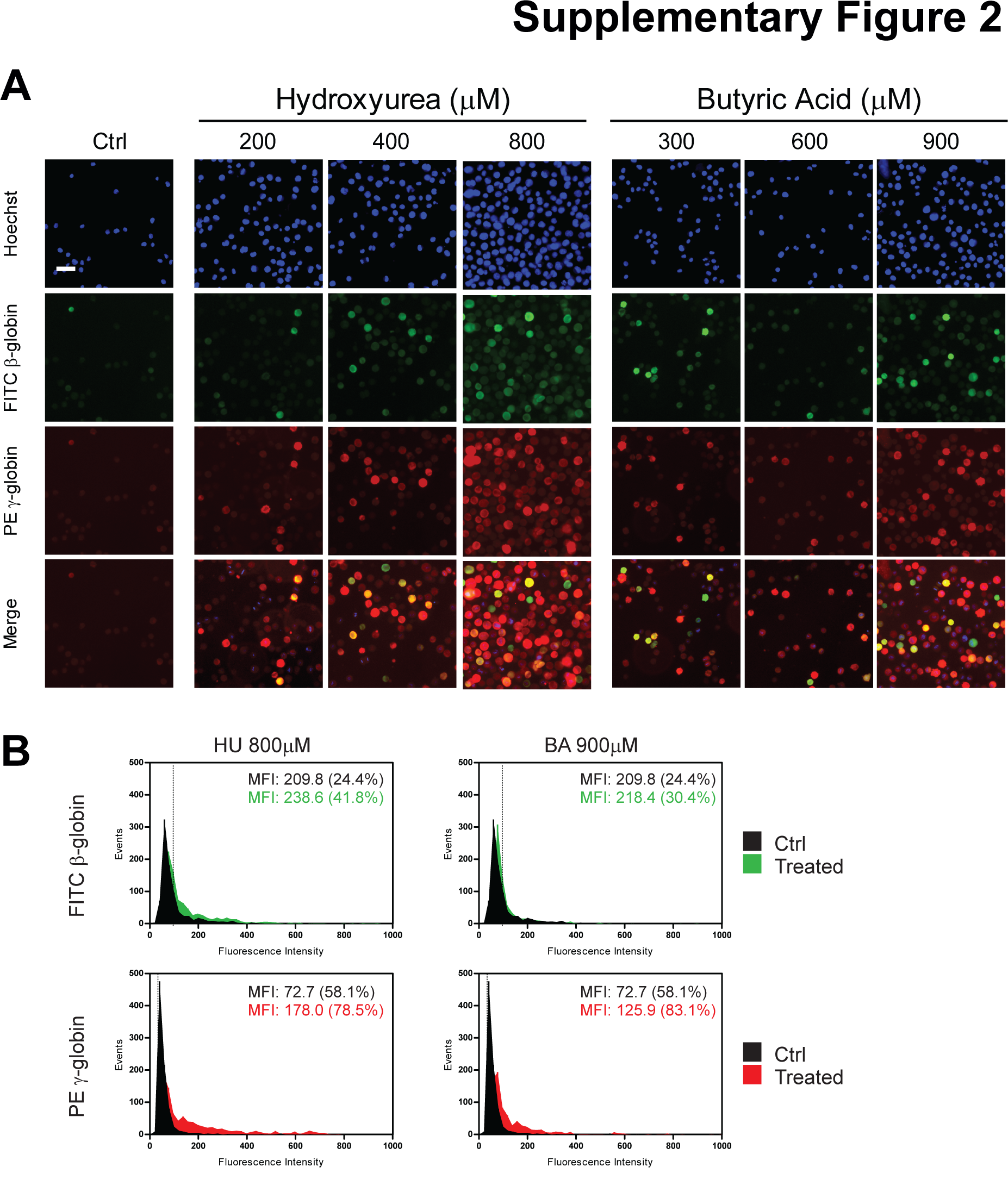

Supplement: S2 Fig — A) Representative ArrayScan pictures of β-K562 cells treated with increasing doses of HU and BA (n≥3). Bar = 50μm. B) Fluorescence intensity plots to better visualize the changes in mean fluorescence intensity (MFI) of stained cells upon drugs treatment. Y axis: number of events (cells); X axis: fluorescence intensity for β-globin signal (upper panels) or γ-globin signal (lower panels), respectively. Green/Red curves: treated cells. Black curve: untreated cells. The vertical dotted line within each panel corresponds to the threshold set in Fig 2A. The MFI and the percentage of positive cells (%) are indicated within each panel. (TIF) [file pone.0141083.s002.tif]

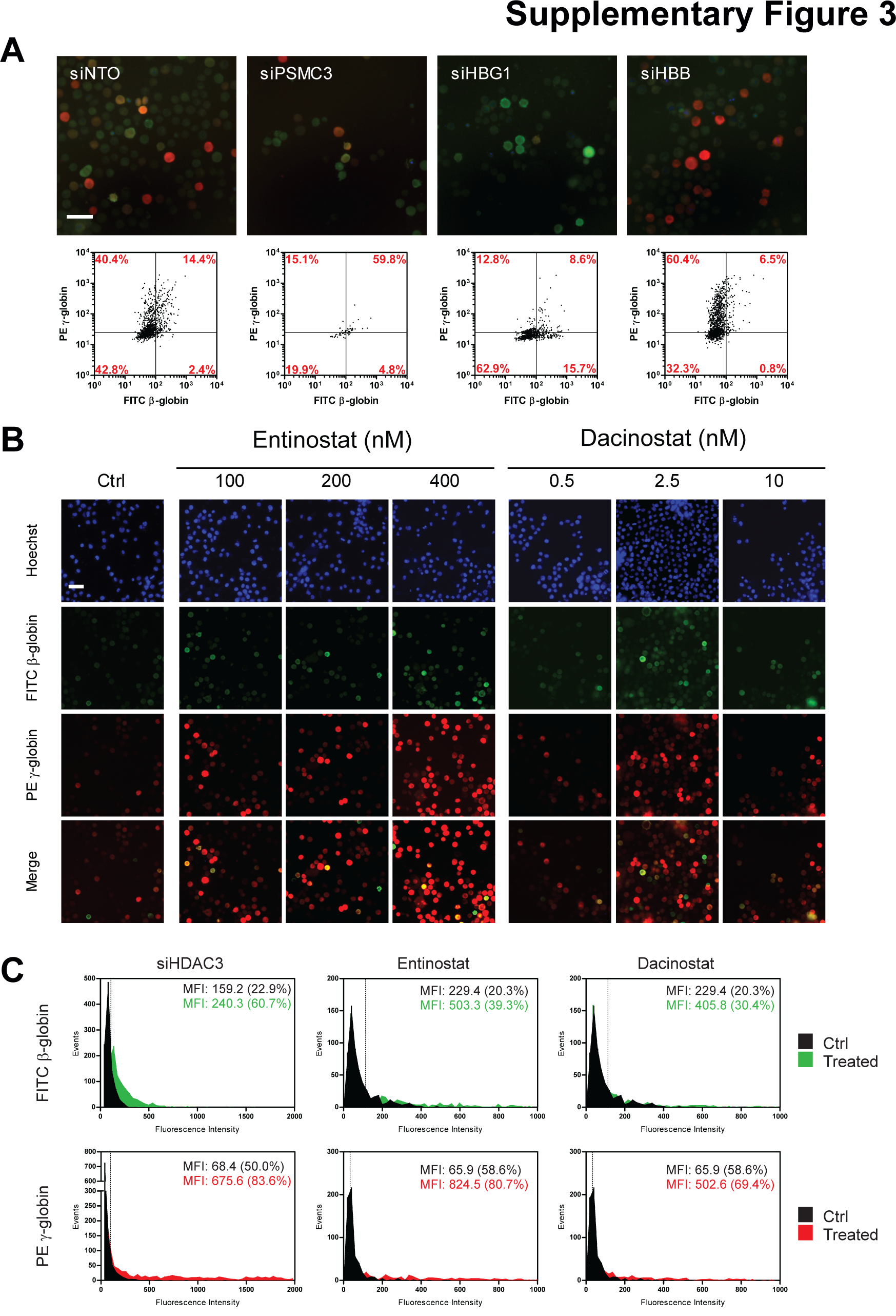

Supplement: S3 Fig — A) Cells were transfected with a non-targeting oligo (siNTO) as negative control and with a siRNA directed to PSMC3 as positive transfection control. As further control, siRNAs targeting γ- and β-globins greatly reduced the corresponding globins chains. For each gene, two siRNAs were tested, with two technical replicates (immunofluorescence images of representative experiments are shown). Bar = 50μm. Scatter plots are provided for each immunofluorescence image (n = 2). B) Representative ArrayScan pictures of β-K562 cells treated with increasing doses of entinostat and dacinostat. C) MFI plots as in S2 Fig. (TIF) [file pone.0141083.s003.tif]

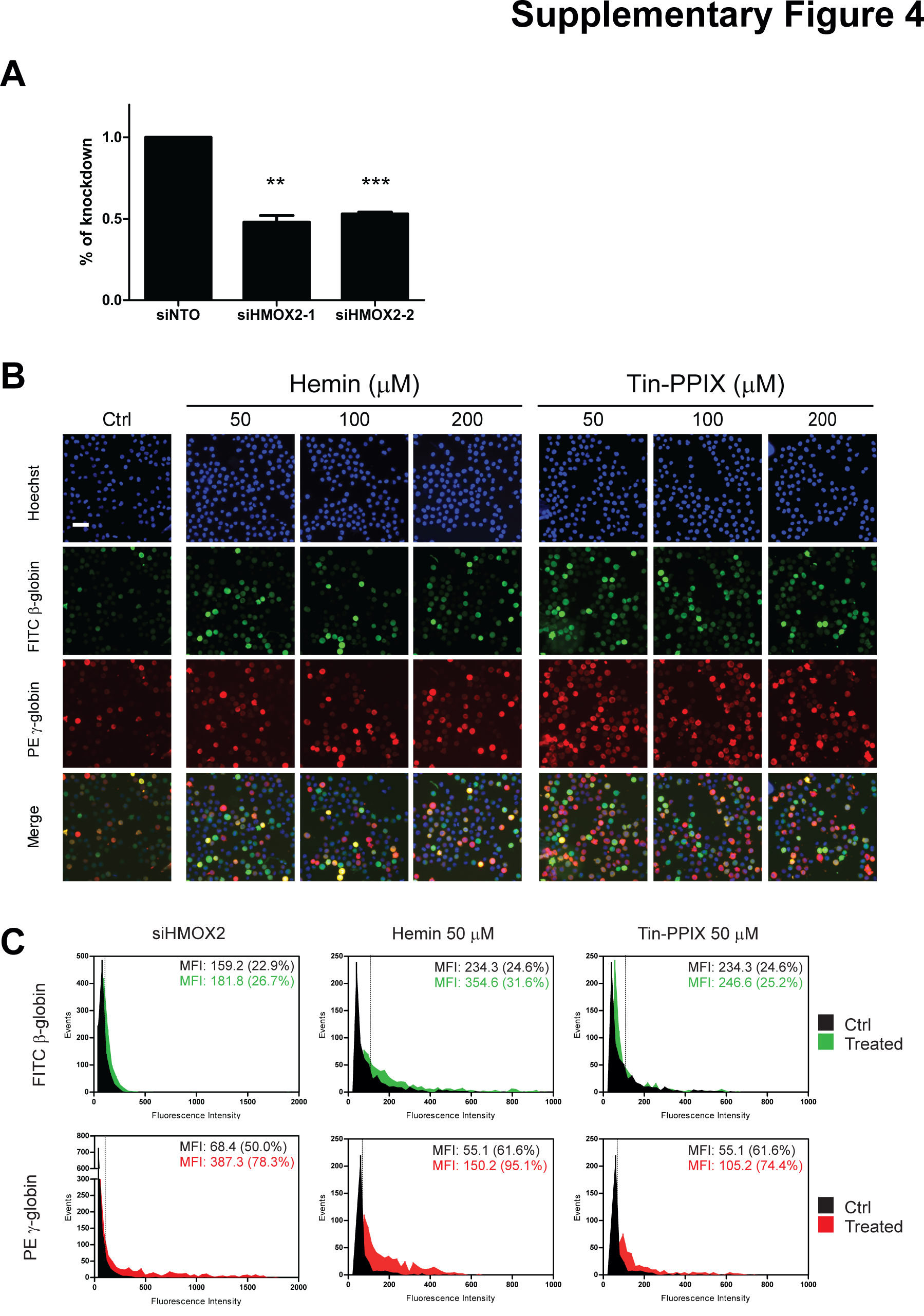

Supplement: S4 Fig — A) HMOX2 knockdown: RTqPCR on cells transfected with a non targeting oligo (siNTO) and with two independent siRNA directed to HMOX2. B) Representative ArrayScan pictures of β-K562 cells treated with increasing doses of Hemin and or Tin-PPIX. Bar = 50μm. C) MFI plots as in S2 Fig. (TIF) [file pone.0141083.s004.tif]
