# Supplementary material for: A Novel High-Content Immunofluorescence Assay as a Tool to Identify at the Single Cell Level γ-Globin Inducing Compounds
Source: PLoS One. 2015 Oct 28;10(10):e0141083. doi: 10.1371/journal.pone.0141083 (PMC4624791; doi:10.1371/journal.pone.0141083)
Supplement: S1 File — (PDF) [file pone.0141083.s005.pdf]

S1 File. siRNAs oligonucleotide detailed transfection method.

β-K562 cells were transfected with siRNA oligonucleotides obtained from the human Silencer Select Druggable Genome siRNA Library V4 (Ambion). For all genes analysed, two different siRNAs were tested. For siRNA showing a promising result, transfections were performed in triplicates. In detail, siRNA oligonucleotides were dissolved in Opti-MEM® I medium (Invitrogen) at a concentration of 120 nM and gently mixed with an equal volume of Opti-MEM® I containing 0.5% v/v lipofectamine® RNAiMAX (Invitrogen). 50 μl/well of transfection mixture was transferred to U-bottom 96-well plates (Greiner Bio-One, Germany) containing 100 μl/well of cells in growth medium at a density of 1500 cells/ml. Final siRNA oligos concentration was set to 20 nM. Plates were incubated for 7 days before high-content analysis (and RTqPCR analysis, when carried out). Cell transfection was done with at least two technical replicates per experiment by using at least two targeting siRNA per gene.
