# Supplementary material for: A Novel High-Content Immunofluorescence Assay as a Tool to Identify at the Single Cell Level γ-Globin Inducing Compounds
Source: PLoS One. 2015 Oct 28;10(10):e0141083. doi: 10.1371/journal.pone.0141083 (PMC4624791; doi:10.1371/journal.pone.0141083)
Supplement: S1 Table — (PDF) [file pone.0141083.s006.pdf]

S1 Table. Chemicals and antibodies

Chemical, antibody:	Catalog number, company:	Note:
Hydroxyurea	H8627, Sigma-Aldrich	
Hemin	H5533, Sigma-Aldrich	*
Entinostat (LAQ824)	*S1095, Selleck Chem*	
Dacinostat (MS-275)	*S1053, Selleck Chem*	
Butyric Acid	*B103500, Sigma-Aldrich*	
Triton^{TM} X-100	X100, Sigma-Aldrich	
Hoechst 33342	B2261, Sigma-Aldrich	
Formaldehyde solution	F1635, Sigma-Aldrich	
Blotting-grade blocker (dry milk)	170-6404, Bio-Rad Laboratories	
Imatinib mesylate	S1026, Selleck Chemicals	
Dasatinib	S1021, Selleck Chemicals	
Doxorubicin	S1208, Selleck Chemicals	
Fetal calf serum (FCS)	EC S0170L, Euroclone	
Phosphate-buffered saline (PBS)	20012-068, Gibco Life Technologies	
RPMI Medium 1640	61870-044, Gibco Life Technologies	
Opti-MEM® I medium	31985-054, Gibco Life Technologies	
PenStrep antibiotics solution	15140-122, Gibco Life Technologies	
Tin protoporphyrin IX	sc-203452, Santa Cruz Biotechnology	*
Hemoglobin γ antibody PE	sc-21756, Santa Cruz Biotechnology	Clone: 51-7
hemoglobin β antibody FITC	sc-21757, Santa Cruz Biotechnology	Clone: 37-8
APC anti-human CD235ab	306608, Biolegend	Clone: HIR2
FITC-conjugated mouse IgG_1	555748, BD Biosciences Pharmingen	Clone: MOPC-21
PE-conjugated mouse IgG_1	551436, BD Biosciences Pharmingen	Clone: MOPC-21

*** Hemin and Tin protoporphyrin IX were dissolved in 1M KOH, pH was adjusted to 7.5 with HCl, and the solution was diluted with Tris-HCl at pH 7.5.**
