# Supplementary material for: A Novel High-Content Immunofluorescence Assay as a Tool to Identify at the Single Cell Level γ-Globin Inducing Compounds
Source: PLoS One. 2015 Oct 28;10(10):e0141083. doi: 10.1371/journal.pone.0141083 (PMC4624791; doi:10.1371/journal.pone.0141083)
Supplement: S2 Table — (PDF) [file pone.0141083.s007.pdf]

S2 Table. List of siRNA oligos

siRNA oligo	Batch	Sense sequence	Antisense sequence
NTO	Synthesized in house	UGGUUUACAUGUCGACUAAtt	UUAGUCGACAUGUAAACCAtt
PSMC3	Synthesized in house	CGGCUGAAGUGCGCAAUAAtt	UUAUUGCGCACUUCAGCCGtt
HBB (oligo 1)	SIRNA_ASO0DT9C	GAAAGUGCUCGGUGCCUUUtt	AAAGGCACCGAGCACUUUCtt
HBB (oligo 2)	SIRNA_ASO0DTBP	AGGUGAACGUGGAUGAAGUtt	ACUUCAUCCACGUUCACCUtg
HBG1 (oligo 1)	SIRNA_ASO0DT70	GUCUACCCAUGGACCCAGAtt	UCUGGGUCCAUGGGUAGACaa
HBG1 (oligo 2)	SIRNA_ASO0F7U8	UGACCGUUUUGGCAAUCCAtt	UGGAUUGCCAAAACGGUCAcc
HMOX2 (oligo 1)	SIRNA_ASO0DTAD	ACAUGCAGAUAUUCAAUGAtt	UCAUUGAAUAUCUGCAUGUta
HMOX2 (oligo 2)	SIRNA_ASO0DTCP	GGAAAGGAGACAUGCGUAAtt	UUACGCAUGUCUCCUUUCCca
HDAC3 (oligo 1)	SIRNA_ASO0DIV6	GAGCUUCAAUAUCCCUCUAtt	UAGAGGGAUAUUGAAGCUCtt
HDAC3 (oligo 2)	SIRNA_ASO0DIV7	CCAAGAGUCUUAAUGCCUUtt	AAGGCAUUAAGACUCUUGGtg
