# Supplementary material for: A Novel High-Content Immunofluorescence Assay as a Tool to Identify at the Single Cell Level γ-Globin Inducing Compounds
Source: PLoS One. 2015 Oct 28;10(10):e0141083. doi: 10.1371/journal.pone.0141083 (PMC4624791; doi:10.1371/journal.pone.0141083)
Supplement: S3 Table — (PDF) [file pone.0141083.s008.pdf]

**S3 Table. List of primers used for RTqPCR**

| Primers | Fwd sequence | Rev sequence |
|---|---|---|
| GAPDH | 5'ACGGATTTGGTCGTATTGGG | 5'TGATTTTGGAGGGATCTCGC |
| α globin | 5'GAGGCCCTGGAGAGGATGTTCC | 5'ACAGCGCGTTGGGCATGTCGTC |
| β globin | 5'TACATTTGCTTCTGACACAAC | 5'ACAGATCCCCAAAGGAC |
| γ globin | 5'CTTCAAGCTCCTGGGAAATGT | 5'GCAGAATAAAGCCTACCTTGAAAG |
| ε globin | 5'GCCTGTGGAGCAAGATGAAT | 5'GCGGGCTTGAGGTTGT |
| Hmox2 | 5'GATGGGTTCCCTGTACACGA | 5'CCATAGCTGTTCGGAAGGGA |
