# Supplementary material for: A Novel High-Content Immunofluorescence Assay as a Tool to Identify at the Single Cell Level γ-Globin Inducing Compounds
Source: PLoS One. 2015 Oct 28;10(10):e0141083. doi: 10.1371/journal.pone.0141083 (PMC4624791; doi:10.1371/journal.pone.0141083)
Supplement: S4 Table — (PDF) [file pone.0141083.s009.pdf]

S4 Table. List of genes tested by siRNA-mediated knockdown and selected from the Ambion-library on the basis of their possible role in erythropoiesis as inferred from literature and public databases.

Gene symbol	RefSeq (mRNA)	Full gene name
BAP1	NM_004656	BRCA1 associated protein-1 (ubiquitin carboxy-terminal hydrolase)
BCL11A	NM_138553	B-cell CLL/lymphoma 11A (zinc finger protein)
BCL11B	NM_022898	B-cell CLL/lymphoma 11B (zinc finger protein)
BCL2L14	NM_030766	BCL2-like 14 (apoptosis facilitator)
BHLHB2	NM_003670	basic helix-loop-helix domain containing, class B, 2
CARM1	NM_199141	coactivator-associated arginine methyltransferase 1
CCNK	NM_003858	cyclin K
CCNL1	NM_020307	cyclin L1
CDKN1B	NM_004064	cyclin-dependent kinase inhibitor 1B (p27, Kip1)
CHD2	NM_001042572	chromodomain helicase DNA binding protein 2
CHD4	NM_001273	chromodomain helicase DNA binding protein 4
CHD5	NM_015557	chromodomain helicase DNA binding protein 5
CHD6	NM_032221	chromodomain helicase DNA binding protein 6
CUX1	NP_001189472	cut-like homeobox 1
DNM2	NM_004945	dynamin 2
DNMT1	NM_001379	DNA (cytosine-5-)-methyltransferase 1
EIF4A1	NM_001416	eukaryotic translation initiation factor 4A, isoform 1
EIF4E2	NM_004846	eukaryotic translation initiation factor 4E family member 2
EIF4G1	NM_004953	eukaryotic translation initiation factor 4 gamma, 1
FOXA2	NM_153675	forkhead box A2
FOXA3	NM_004497	forkhead box A3
HBA1	NM_000558	hemoglobin, alpha 1
HBB	NM_000518	hemoglobin, beta
HBD	NM_000519	hemoglobin, delta
HBE1	NM_005330	hemoglobin, epsilon 1
HBG1	NM_000559	hemoglobin, gamma A
HBM	NM_001003938	hemoglobin, mu
HBQ1	NM_005331	hemoglobin, theta 1
HBS1L	NM_006620	HBS1-like (S. cerevisiae)
HDAC1	NM_004964	histone deacetylase 1
HDAC2	NM_001527	histone deacetylase 2
HDAC3	NM_003883	histone deacetylase 3
HMOX1	NM_002133	heme oxygenase (decycling) 1
HMOX2	NM_002134	heme oxygenase (decycling) 2
KDM1A	NM_015013	amine oxidase (flavin containing) domain 2
LIMA1	NM_016357	LIM domain and actin binding 1
MATR3	NM_018834	matrin 3

MBD2	NM_003927	methyl-CpG binding domain protein 2
MBD3	NM_003926	methyl-CpG binding domain protein 3
MTA1	NM_004689	metastasis associated 1
MTA2	NM_004739	metastasis associated 1 family, member 2
MTA3	NM_020744	metastasis associated 1 family, member 3
MYC	NM_002467	v-myc myelocytomatosis viral oncogene homolog (avian)
NCOA5	NM_020967	nuclear receptor coactivator 5
NR2C1	NM_001032287,M_003297	nuclear receptor subfamily 2, group C, member 1
NR2C2	NM_003298	nuclear receptor subfamily 2, group C, member 2
NR2F1	NM_005654	nuclear receptor subfamily 2, group F, member 1
NR2F2	NM_021005	nuclear receptor subfamily 2, group F, member 2
PHC2	NM_198040	polyhomeotic homolog 2 (Drosophila)
PKP3	XM_001129327	plakophilin 3
PRMT5	NM_006109	protein arginine methyltransferase 5
RBM17	NM_032905	RNA binding motif protein 17
RCOR1	NM_015156	REST corepressor 1
RCOR2	NM_173587	REST corepressor 2
SF1	NM_001178030	splicing factor 1
SMARCA5	NM_003601	SWI/SNF related, matrix associated, actin dependent regulator of chromatin, subfamily a, member 5
SMARCC2	NM_001130420	SWI/SNF related, matrix associated, actin dependent regulator of chromatin, subfamily c, member 2
SOX6	NM_017508	SRY (sex determining region Y)-box 6
SPATS1	NM_145026	spermatogenesis associated, Serine-rich 1
SRF	NM_001292001	serum response factor (c-fos serum response element-binding transcription factor)
SSRP1	NM_003146	structure specific recognition protein 1
SUPT16H	NM_007192	suppressor of Ty 16 homolog (S. cerevisiae)
TRDMT1	NM_004412	aspartic acid methyltransferase 1
TRIP4	NM_016213	thyroid hormone receptor interactor 4
TTN	NM_001256850	titin
UGT1A1	NM_000463	UDP glucuronosyltransferase 1 family, polypeptide A1
UGT1A10	NM_019075	UDP glucuronosyltransferase 1 family, polypeptide A10
VEZF1	NM_007146	vascular endothelial zinc finger 1
VIL1	NM_007127	villin 1
VIM	NM_003380	vimentin
